# Glare prediction and mechanism of adaptation following implantation of hydrophilic and hydrophobic intraocular lenses

**DOI:** 10.3389/fopht.2024.1310468

**Published:** 2024-04-25

**Authors:** Gurpreet K. Bhogal-Bhamra, Maana Aujla, Sai Kolli, Amy L. Sheppard, James S. Wolffsohn

**Affiliations:** ^1^ Ophthalmic Research Group, Aston University, Birmingham, United Kingdom; ^2^ University Hospitals Trust, Ophthalmology, Queen Elizabeth Hospital, Birmingham, United Kingdom

**Keywords:** intraocular lens (IOLs), cataracts, dysphotopsia, glare, cataract surgery

## Abstract

**Purpose:**

Glare is a known side effect of intraocular lens (IOL) implantation, affected principally by IOL material and optics, although it is reported subjectively to decrease in impact with time. However, little objective data have been published on changes over time, how these relate to subjective reports, and whether those who will report greater glare symptoms can be predicted prior to IOL implantation.

**Methods:**

A total of 32 patients (aged 72.4 ± 8.0 years) with healthy eyes were implanted bilaterally with hydrophilic 600s (Rayner, Worthing, UK) or hydrophobic Acrysof (Alcon, Texas, USA) acrylic IOLs (*n* = 16 each, randomly assigned). Each patient reported their dysphotopsia symptoms subjectively using the validated forced choice photographic questionnaire for photic phenomena, and halo size resulting from a bright light in a dark environment was quantified objectively in eight orientations using the Aston Halometer. Assessment was performed binocularly pre-operatively and at 1, 2, 3, and 4 weeks after IOL implantation.

**Setting:**

The study was carried out at the National Health Service Ophthalmology Department, Queen Elizabeth Hospital, Birmingham, UK.

**Results:**

Visual acuity (average 0.37 ± 0.26 logMAR) did not correlate with subjective glare (*r* = 0.184, *p* = 0.494) or objective glare (*r* = 0.294, *p* = 0.270) pre-surgery. Objective halo size (*F* = 112.781, *p* < 0.001) decreased with cataract removal and IOL implantation and continued to decreased over the month after surgery. Subjective dysphotopsia complaints (*p* < 0.001) were also greater pre-surgery, but did not change thereafter (*p* = 0.228). In neither case was there a difference with IOL material (*p* > 0.05). It was not possible to predict post-surgery dysphotopsia from symptoms or a ratio of symptoms to halo size pre-surgery (*p* > 0.05).

**Conclusions:**

Subjective dysphotopsia and objective halos caused by cataracts are greatly reduced by implantation of IOL after cataract removal causing few perceivable symptoms. However, objective measures are able to quantify a further reduction in light scatter over the first month post-IOL implantation, suggesting that any subjective effects over this period are due to the healing process and not due to neuroadaptation.

## Introduction

Undesirable optical phenomena such as negative and positive dysphotopsias are known side effects following modern cataract surgery (1–3) and are the primary causes of post-surgical dissatisfaction in a normal pseudophakic population (4, 5). Negative dysphotopsia is defined as the perception of a shadow obscuring the temporal field of vision, while positive dysphotopsia is characterized as halos, arcs, or streaks around point light sources (2, 6). The prevalence of positive dysphotopsia is reported to range from as low as 1.5% to as high as 67%, with most authors identifying more moderate values of 12% to 35% (1, 7, 8). Negative dysphotopsia is less common and estimated to occur in only 0.5% to 2.4% of patients (8, 9). A Cochrane review of multifocal IOLs found that photic phenomena are 3.5 times more likely to occur with multifocal IOLs than with monofocal IOLs (10). However, Souza et al. (11) reported values of 13% and 20% for glare and halos, respectively, in eyes fitted with monofocals; a more recent study demonstrated that the perception was not detected through straylight, but was partially correlated with halo obscuration size (12).

In the majority of cases, subjective dysphotopsia resolves or diminishes over time (13, 14), although it can be reported more than a year after cataract surgery (15). It has been suggested that this is due to neuroadaptation (16), although in 0.2% to 1% of pseudophakic patients, severe symptoms will persist (6) and additional surgery may be required.

There is currently no widely accepted management strategy for positive dysphotopsia (17). If severe symptoms persist after 4 to 6 weeks, intraocular lens (IOL) exchange may be considered; however, this is considered a last resort (18). The IOL may develop a strong adherence to the capsule, making it difficult to dissect it from the capsular bag (19). Therefore, it is important to be able to distinguish those individuals who are more likely to encounter these problems prior to surgery.

Dysphotopsias were virtually unknown when polymethyl methacrylate (PMMA) was the IOL material of choice (20), although at that time, designs were all monofocal, which have less incidence of photic effects. The inability of relatively stiff PMMA IOLs to fold, requiring a large incision during surgery, and the high rate of posterior capsular opacification (PCO) due to the round edge design have resulted in these lenses rarely being used today (21). However, acrylic lens materials may increase the incidence of dysphotopsia (20). IOLs of PMMA and silicone with rounded edges, along with square-edge acrylic IOLs with non-reflective surfaces, appear less likely to cause clinically significant pseudophakic dysphotopsia (6). Monocular straylight is lower with a hydrophilic IOL than either a hydrophobic or PMMA IOL, but there was no change from 1 week to 1 month after surgery and the differences were noted to be minimal (22, 23). Akman et al. (24) had a similar finding in a retrospective study with a test that assessed contrast sensitivity with and without an annular glare source.

There are limited published data regarding changes in objective and subjective dysphotopsia measures in response to cataract surgery. Numerous studies have reported post-operative subjective effects but are lacking pre-operative measures (to assess predictive ability) and objective assessments and are usually not examined on a longitudinal basis (15). Therefore, the aim of this prospective study was to determine how cataract surgery impacts objective and subjective photic effects immediately after surgery and neuroadaptation, and how the ratio of subjective glare to objective glare assessment prior to surgery might predict patients who suffer from dysphotopsia post-implantation.

## Methods

This prospective study included patients undergoing routine cataract surgery and implantation of hydrophilic acrylic (Rayner 600S, Worthing, UK) and hydrophobic acrylic (Alcon Acrysof, Geneva, Switzerland) monofocal IOLs. All study procedures were performed in the Ophthalmology Outpatients clinic at Queen Elizabeth Hospital, Birmingham, United Kingdom. The study was conducted in accordance with the tenets of the Declaration of Helsinki and received a favorable ethical opinion from the Aston University and University Hospitals Birmingham ethics committees. After receiving an explanation of the nature and possible consequences of the study, all subjects gave their written informed consent to take part.

A total of 32 patients with no previous ocular complications and with bilateral visually significant cataract scheduled for routine phacoemulsification cataract surgery and IOL implantation were enrolled in the study. Exclusion criteria also included the potential for best-corrected visual acuity that is worse than 0.30 logMAR; partial or total paralysis; Parkinson’s disease, cerebrovascular accident, or other conditions that could affect the results of the study; physical and/or mental conditions that could hinder participation; and a history of using drugs that are known to affect visual function measures. All patients had cataract surgery under topical anesthesia performed by the same experienced surgeon, in both eyes. A standard sutureless microincision phacoemulsification technique was used. The IOL (randomly selected) was implanted in the capsular bag with a single-use injection system. Post-operatively, topical therapy included a standard combination of antibiotic and steroidal agents.

At the pre-operative assessment, participants were examined to judge their suitability for cataract surgery. A slit-lamp examination of the anterior segment and fundoscopy of the optic nerve head and macular region were performed. The condition of the lens opacification was scored using the LOCS III system (25). Unaided vision or visual acuity in their current spectacles or with pinhole was recorded using a logMAR chart.

Objective measures of dysphotopsia were acquired using the Aston Halometer positioned at 2 m from the patient in a dark room. The Aston Halometer consists of an iPad Air (Apple Inc., California, USA) fitted with a bespoke sleeve with a rotatable rod to position a light-emitting diode on the center. The software app allows 0.3 logMAR, 50% contrast letters to be moved eccentrically with detection angle scored as when the patient can report at least two out of three randomized capital letter presentations correctly (26). An iPhone 5S (Apple Inc, California, USA) acted as the remote to control the movement and randomization of the letter. Halo radius was measured in eight directions 45° apart, binocularly. The PIPP images used to subjectively grade dysphotopsia were also presented on the iPad through an app with patient selecting the type of dysphotopsia experienced and grading the severity of these through an image-guided grading scale of a four-point scale (27).

Subjective and objective dysphotopsia measures were assessed prior to surgery and weekly over the first month post-surgery. Measures were taken binocularly to depict real-world viewing.

### Statistical analysis

Power calculations, made using GPower (version 3.1.9.2), showed that a total of 32 participants were required to enable a repeated-measures ANOVA to detect statistically significant effect size (0.25) at the 5% significance level (α = 0.05) with 95% power. All statistical tests were performed using SPSS statistical software (v25, IBM, Armonk, New York, US). The one-sample Kolmogorov–Smirnov test was used to determine if results from each measurement followed a normal distribution, which confirmed that the objective data were not significantly different from a normal distribution (*p* > 0.05). To track changes in objective measures over time, a parametric repeated-measures ANOVA was used, and for subjective measures, a non-parametric Friedman repeated-measures ANOVA on Ranks was used. Associations were assessed using Spearman’s rank correlation. In all cases, a *p*-value of <0.05 was considered statistically significant.

## Results

All patients underwent uncomplicated phacoemulsification extraction and IOL implantation. The IOL groups were similar in age (71.8 ± 7.4 vs. 73.1 ± 8.8, *p* = 0.793) and sex (6 male patients in each). Visual acuity (average 0.37 ± 0.26 logMAR) did not correlate with subjective glare (*r* = 0.184, *p* = 0.494) or objective glare (*r* = 0.294, *p* = 0.270) pre-surgery.

**Figure 1:**
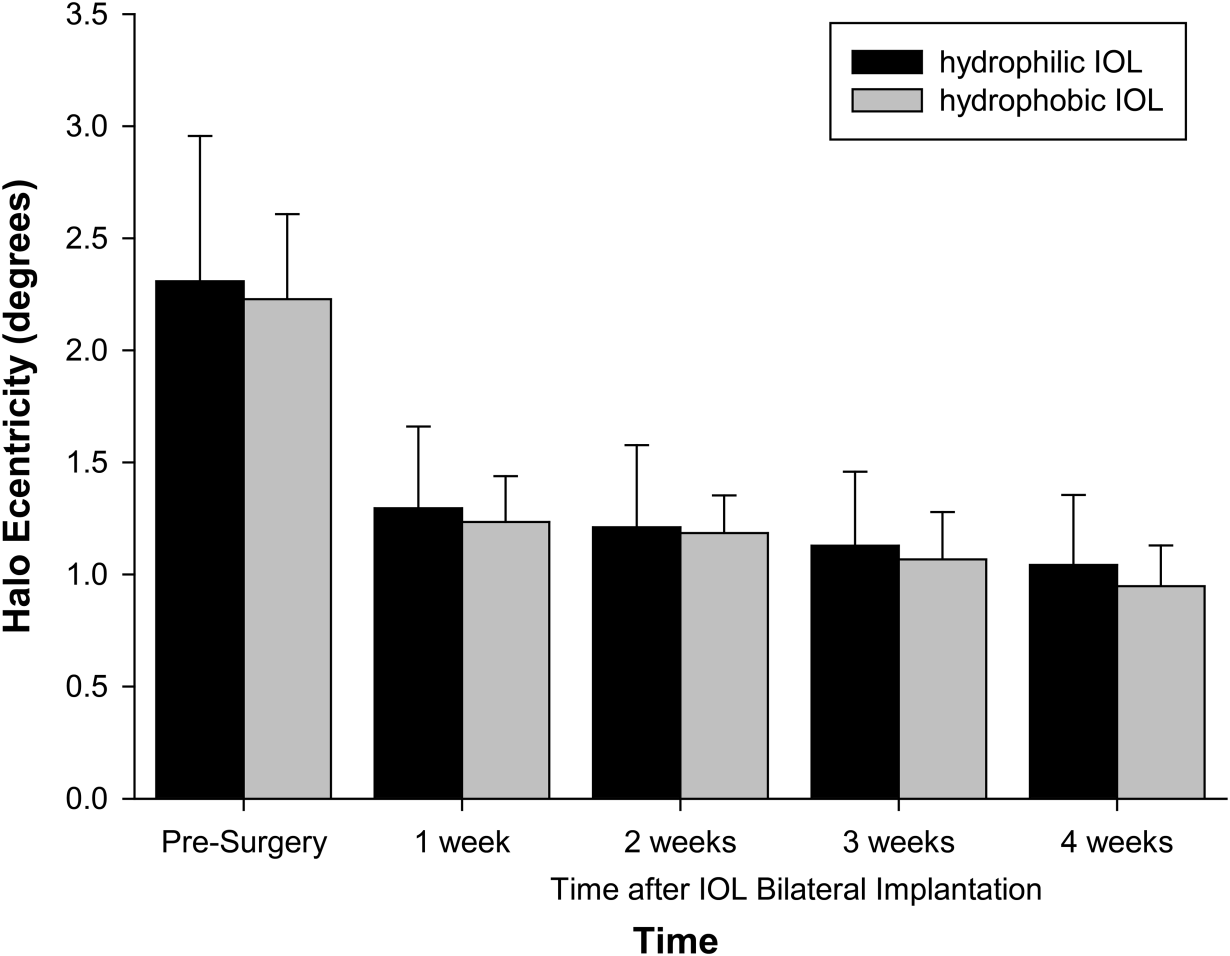
Halo size with IOL material and time after surgery. Error bars = 1 SD. *n* = 16 hydrophilic, *n* = 16 hydrophobic.

Objective halo size was large with cataracts before surgery and decreased with IOL implantation (*F* = 112.781, *p* < 0.001). It continued to decrease after surgery (1–2 weeks: *p* = 0.069; 2–3 weeks: *p* = 0.003; 3–4 weeks: *p* = 0.022). It did not differ with IOL material (*F* = 1.490, *p* = 0.244) (**Figure 1**).

The halo was asymmetric in profile (*F* = 5.734, *p* < 0.001), and this varied with IOL material (*F* = 3.079, *p* = 0.007), but the average maximum difference between the meridians was only 0.15 degrees with the hydrophilic IOLs and 0.08 degrees with the hydrophobic IOLs.

**Figure 2:**
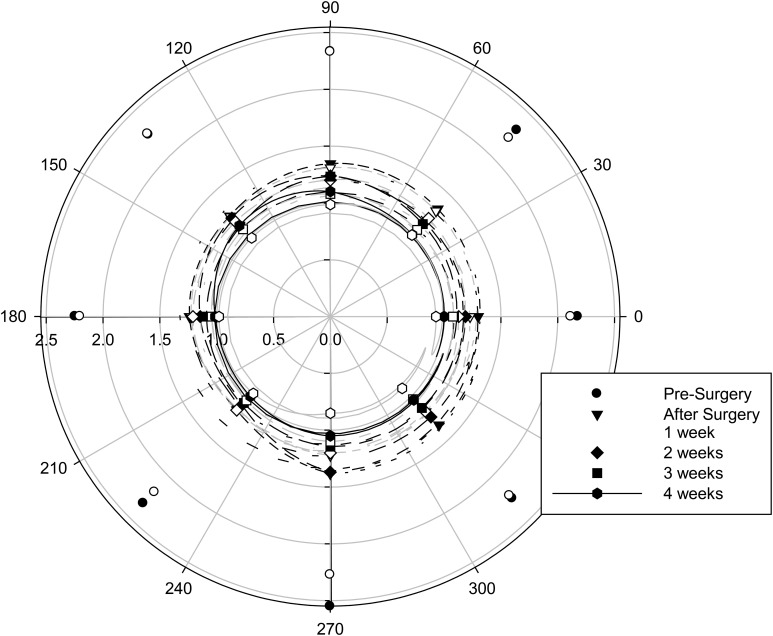
Halo profile with IOL material (filled symbols with black lines, hydrophilic IOLs; open symbols with gray lines, hydrophobic IOLs) and time after surgery. *n* = 16 hydrophilic, *n* = 16 hydrophobic.

The median number of reported dysphotopsia categories reported prior to cataract surgery was 2 with a range of 0–2 (**Figure 2**). Less than 10% of patients reported a ripple effect, halos and stars, plate glare, and bright or dark arcs. The most commonly reported dysphotopsia was night glare reported by 50% of patients and sunburst and central glare reported by 31% before cataract surgery. The summed severity of subjective glare significantly decreased after cataract surgery (*p* < 0.001), but there was no difference with time after surgery (*p* = 0.228). Again, there was no effect of IOL material (*p* = 0.294–0.854).

**Figure 3:**
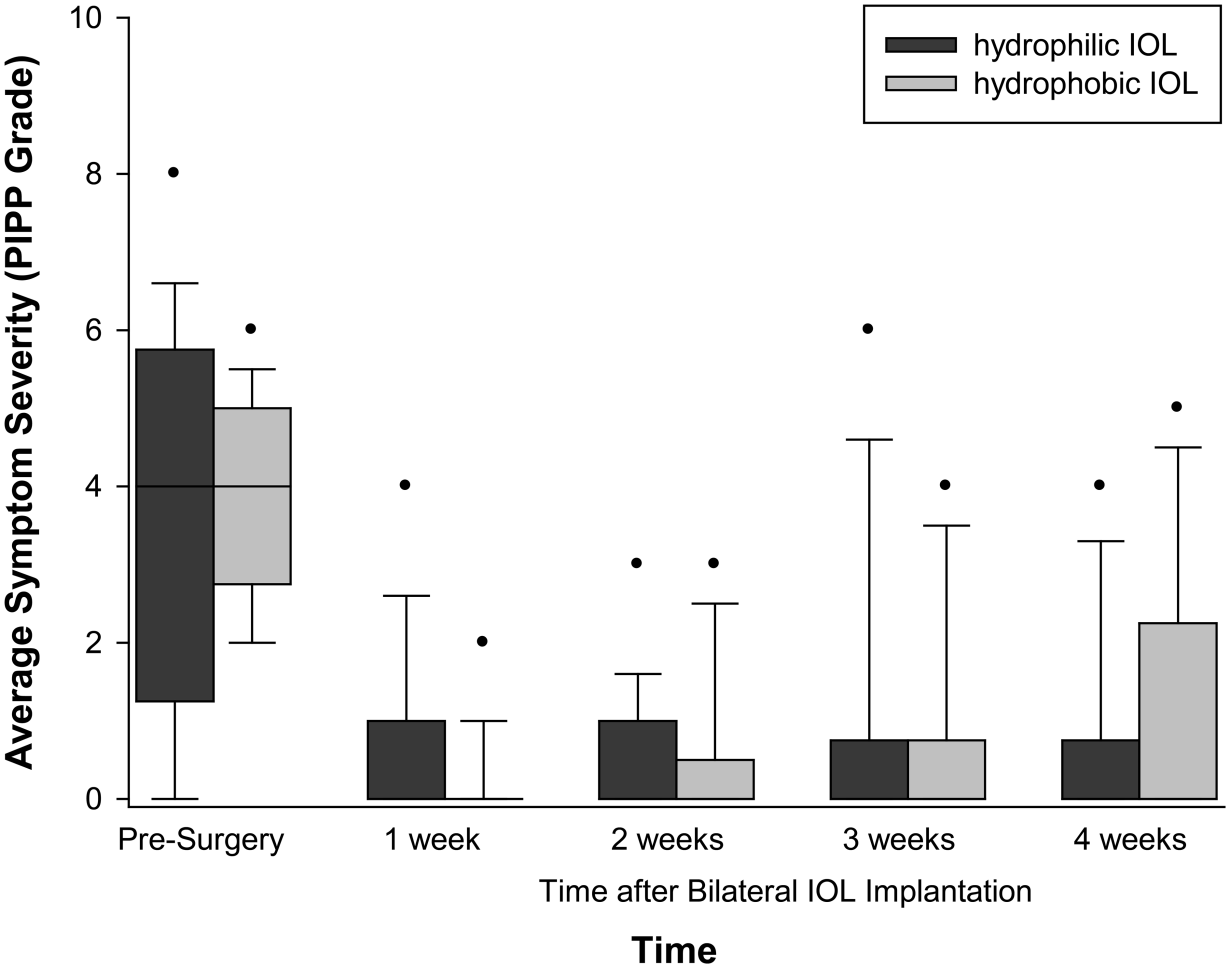
Dysphotopsia symptomology with IOL material and time after surgery. Box = 1 SD, line = median and whiskers 95% confidence interval. *n* = 16 hydrophilic, *n* = 16 hydrophobic.

Objective glare was correlated with the subjectively reported average severity (0.403, *p* = 0.027), but not the number (0.364, *p* = 0.48) of subjective dysphotopsia symptoms before cataract surgery (**Figure 3**).

There was no significant correlation between the ratio of subjective dysphotopsia complaints to objective halo size pre-surgery compared to subjective dysphotopsia complaints at weeks 1–4 after surgery although the effect got larger with time (week 1: *r* = 0.136, *p* = 0.472; week 2: *r* = 0.214, *p* = 0.256; week 3: *r* = 0.226, *p* = 0.230; week 4: *r* = 0.300, *p* = 0.107). However, a similar pattern was seen with subjectively reported glare before and after surgery (week 1: *r* = 0.194, *p* = 0.305; week 2: *r* = 0.240, *p* = 0.201; week 3: *r* = 0.250, *p* = 0.184; week 4: *r* = 0.308, *p* = 0.097).

## Discussion

Modern cataract surgery is extremely successful at improving patients’ vision and quality of life. However, there is a small percentage of patients who remain dissatisfied after the procedure, even with good visual acuity. Dysphotopsia is a chief complaint after an otherwise successful cataract surgery (1, 4, 5). Considering this, few studies have investigated the change in objective and subjective measures of dysphotopsia in response to cataract surgery. While some studies have reported post-operative effects, they are rarely on a longitudinal basis (15) with no pre-operative measures for comparison.

There is some evidence that glare improves after surgery presented by Van den Berg et al. (28), who assessed straylight in pseudophakic eyes, non-cataractous eyes, and cataractous eyes. The cataract eyes had a relatively mild increase in straylight compared to non-cataract eyes. Surprisingly, in pseudophakia, straylight values were better than in the non-cataract group. However, the study did not compare the pre- and post-operative measures in the same participants, but instead compared a group with cataract to a different post-cataract surgery group. The lens starts to change color from colorless at age 20 to 25 years to slight yellow, up to brown at approximately 65 years and above (28). The lens continues to grow throughout life, creating more and more optical distortions (29). The retained anatomic layers of the crystalline lens from the embryonic stage to the adult stage may be one of the causes of light scattering in the eye (30). Hence, a likely reason why pseudophakic participants performed better than the non-cataractous group is that even without the presence of significant cataracts, there will be some degree of normal age-related scattering occurring compared to the colorless IOL.

In this longitudinal study, both objective and subjective glare decreased with cataract surgery, as expected. There was a systematic decrease in objective glare, where the subjective reporting of glare was low, but more variable. The decrease in objective glare is likely to be related to wound healing (perhaps explaining the asymmetry) and suggests that previous reports of glare reducing with time after surgery (13) are due to optical changes rather than neuroadaptation. Despite previous reports that acrylic lens materials may increase the incidence of dysphotopia (20), that was not the case in this study. The participants were randomly assigned to the IOL material given, and although pupil size can affect dysphotopsia, it was similar between IOL material groups. The patients had no observed corneal or IOL opacities post-implantation.

Pre-operatively, there was a lack of relationship between VA and measured dysphotopsia, both objective halo size and subjective grade. This finding is concurrent with several previously published studies reporting that the two measures are independent of each other, with dysphotopsia often present despite excellent visual acuity (4, 5, 28). The proposed glare effect ratio (subjective glare grade divided by objective halo size) was not more predictive than the preoperative subjective dysphotopsia reported alone in identifying those patients with a greater propensity to be bothered subjectively by less objective glare than other people. These individuals may need additional counseling prior to surgery and/or recommendation of IOLs with less complex optics; thus, they need to be identified in advance before surgery. The findings may relate to the low symptom rate (~20% of participants) after surgery, and in patients implanted with multifocal IOLs for whom dyphotopsia is more commonly reported.

To conclude, this study has shown that both objective and subjective measures of dysphotopsia improve significantly with uncomplicated cataract surgery. Objective halo size is sensitive enough to detect further improvements over the month most after the surgery.

## Data availability statement

The raw data supporting the conclusions of this article will be made available by the authors, without undue reservation.

## Ethics statement

The studies involving humans were approved by West Midlands - South Birmingham Research NHS Ethics Committee. The studies were conducted in accordance with the local legislation and institutional requirements. The participants provided their written informed consent to participate in this study.

## Author contributions

GB-B: Investigation, Methodology, Validation, Writing – original draft. MA: Investigation, Methodology, Validation, Writing – original draft. SK: Conceptualization, Investigation, Methodology, Validation, Writing – review & editing. AS: Conceptualization, Methodology, Validation, Writing – review & editing. JW: Conceptualization, Data curation, Funding acquisition, Methodology, Supervision, Writing – review & editing.
